# Supplementary material for: Correlative light and electron microscopy suggests that mutant huntingtin dysregulates the endolysosomal pathway in presymptomatic Huntington’s disease
Source: Acta Neuropathol Commun. 2021 Apr 14;9:70. doi: 10.1186/s40478-021-01172-z (PMC8048291; doi:10.1186/s40478-021-01172-z)
Supplement: Supplementary file 1 — Additional File 1: Supplementary information. [file 40478_2021_1172_MOESM1_ESM.pdf]

Additional File 1: Supplementary information

Correlative light and electron microscopy suggests that mutant huntingtin dysregulates the endolysosomal pathway in presymptomatic Huntington's disease

Ya Zhou[1], Thomas R. Peskett[2,3], Christian Landles[1], John B. Warner[4], Kirupa Sathasivam[1], Edward J. Smith[1], Shu Chen[2], Ron Wetzel[5], Hilal A. Lashuel[4], Gillian P. Bates[1,†*], Helen R. Saibil[2†*]

[1]Huntington's Disease Centre, Department of Neurodegenerative Disease and UK Dementia Research Institute at UCL, Queen Square Institute of Neurology, University College London, London, UK

[2]Institute of Structural and Molecular Biology, Birkbeck College, London, WC1E 7HX, UK

[3]Present address: Institute of Biochemistry, Department of Biology, ETH Zurich, Otto-Stern-Weg 3, 8093 Zurich, Switzerland

[4]Laboratory of Molecular and Chemical Biology of Neurodegeneration, Brain Mind Institute, École Polytechnique Fédérale de Lausanne (EPFL), Lausanne, Switzerland

[5]Department of Structural Biology, University of Pittsburgh School of Medicine, Pittsburgh, PA 15260, USA.

*Correspondence: h.saibil@mail.cryst.bbk.ac.uk (H.R.S.); gillian.bates@ucl.ac.uk (G.P.B)

†These authors contributed equally

Table S1 Overview of the antibodies used in the present study

Antibody	Species	Dilution			Source	Product number
		IF/IHC	WB	TEM		
S830	Sheep polyclonal	1:1000	1:2000	1:20	[1]	N/A
mEM48	Mouse monoclonal	1:100	-	1:20	EMD Millipore	Cat# MAB5374 RRID: AB_10055116
RAB5	Rabbit polyclonal	5 µg/ml	1:1000	-	Abcam	Cat# ab18211 RRID: AB_470264
RAB7	Rabbit monoclonal	1:100	1:1000	-	Abcam	Cat# ab137029 RRID: AB_2629474
RAB11	Mouse	-	1:1000	-	BD Biosciences	Cat# BD 610656 RRID: AB_397983
RAB27A	Mouse monoclonal	1:100	1:500	-	Abcam	Cat# ab55667 RRID: AB_945112
CD63	Rabbit monoclonal	1:100	1:1000	-	Abcam	Cat# ab217345 RRID: AB_2754982
LAMP1	Rat monoclonal	1:100	1:500	-	Abcam	Cat# ab25245 RRID: AB_449893
Cathepsin B	Rabbit monoclonal	1:200	1:1000	-	Abcam	Cat# ab214428 RRID: AB_2848144
Cathepsin D	Rabbit monoclonal	1:200	1:1000	-	Abcam	Cat# ab75852 RRID: AB_1523267
M6PR	Rabbit monoclonal	1 µg/ml	1:1000	-	Abcam	Cat# ab124767 RRID: AB_10974087
ATP5B	Mouse monoclonal	-	1:25000	-	Abcam	Cat# ab14730 RRID: AB_301438
Rat Alexa Fluor 594	Donkey polyclonal	1:1000			Thermo Fisher Scientific	Cat# A-21209 RRID: AB_2535795
Sheep Alexa Fluor 647	Donkey polyclonal	1:1000			Thermo Fisher Scientific	Cat# A-21448 RRID: AB_2535865
Mouse Alexa Fluor Plus 555	Donkey polyclonal	1:1000			Thermo Fisher Scientific	Cat: A32773 RRID: AB_2762848
Goat Alexa Fluor 594	Donkey polyclonal	1:1000			Thermo Fisher Scientific	Cat# A-11058 RRID: AB_2534105
Rabbit Alexa Fluor Plus 555	Donkey polyclonal	1:1000			Thermo Fisher Scientific	Cat# A32794 RRID: AB_2762834
Mouse 20nm gold conjugated	Goat polyclonal			1:50	Abcam	Cat# ab27242 RRID: AB_954469
Sheep 10nm gold conjugated	Rabbit polyclonal			1:50	Abcam	Cat# ab39609 RRID: AB_954445

IF = immunofluorescence; IHC = immunohistochemistry; WB = western blot; TEM = transmission electron microscopy.

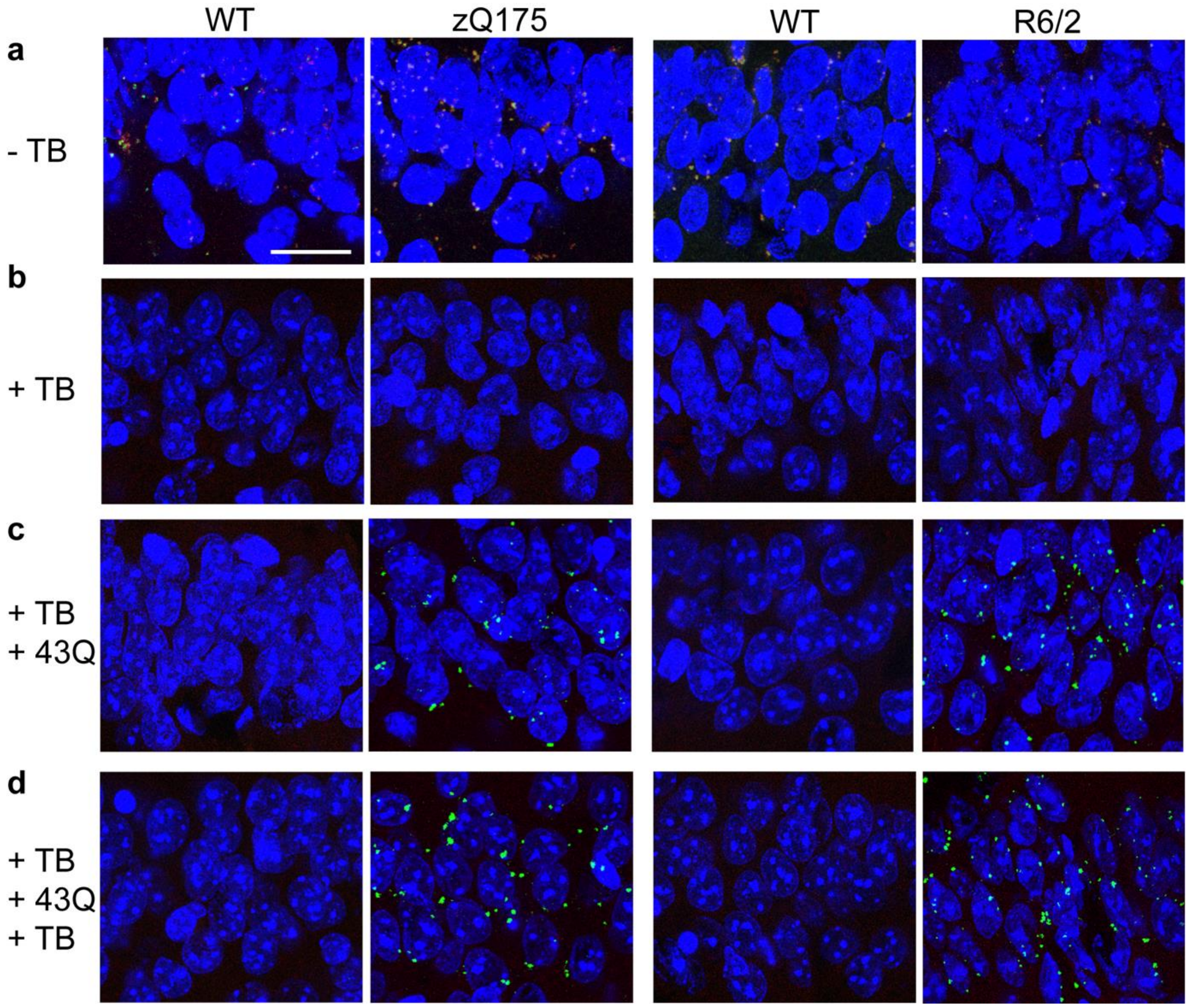

Fig. S1 Trueblack quenches brain tissue autofluorescence, but not the HTT-exon1-43Q fluorescence signal. **a** Without Trueblack treatment, autofluorescent puncta could be observed in brain sections from 6-month-old zQ175 and 12-week-old R6/2 mice and their wild-type littermates under both 488 nm and 561 nm lasers, using the filter settings for Alexa Fluor 488 and Alexa Fluor 568. The same imaging settings were used for all images in this figure, showing signals from both channels plus Hoechst for nuclear counterstaining. **b** After TrueBlack treatment, autofluorescent puncta were quenched in zQ175, R6/2 and wild-type brain sections. **c** When TrueBlack-treated zQ175, R6/2 and wild-type brains sections were incubated with the HTT-exon1-43Q-AF488 peptide, the recruitment signal was only observed on the zQ175 and R6/2 sections. **d** When Trueblack treated sections that had been incubated with the HTT-exon1-43Q peptide were again treated with TrueBlack, no quenching of the recruitment signal was observed. Scale bar: 20 µm. WT = wild-type, TB = Trueblack, 43Q = HTT-exon1-43Q-AF488.

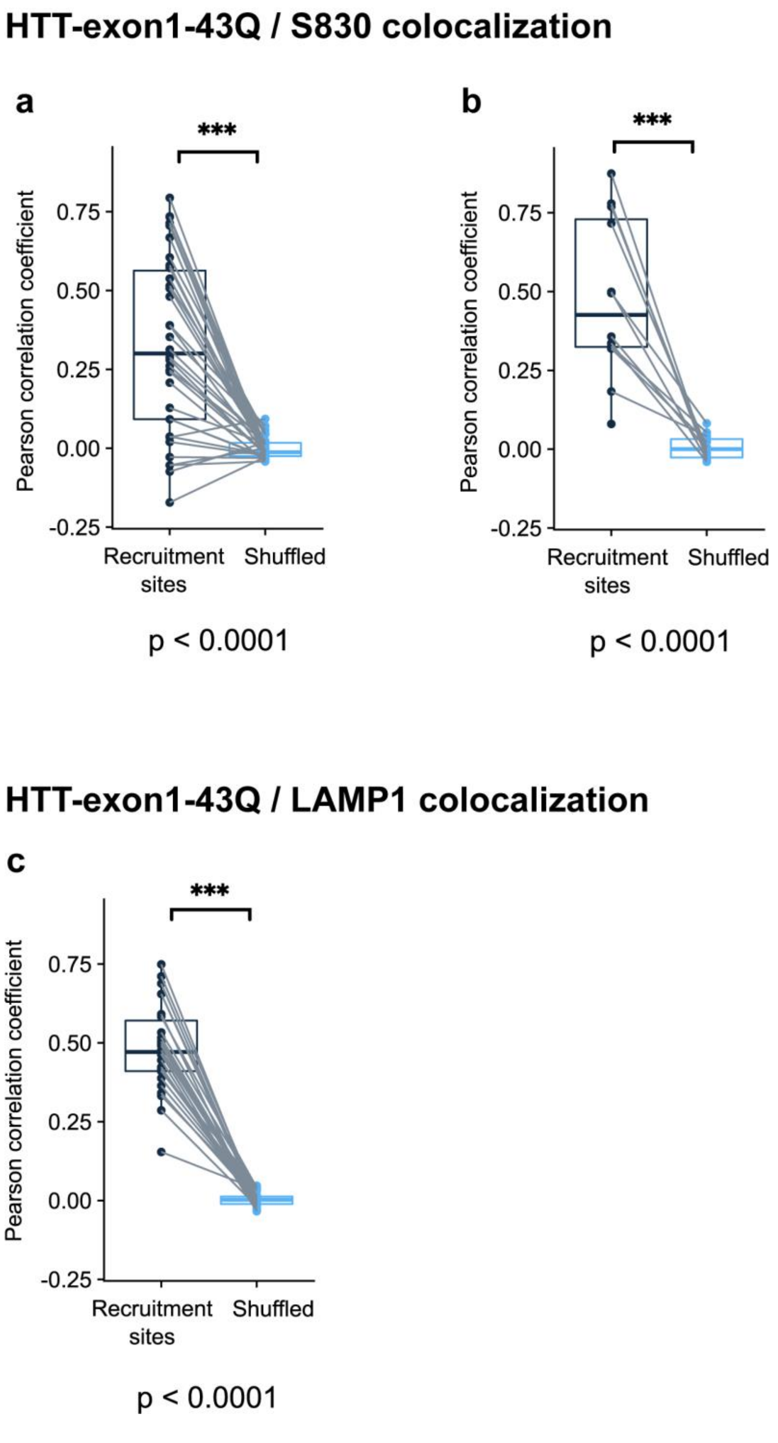

Fig. S2 Colocalization analysis of confocal images. **a** Pearson correlation coefficient was calculated using the HTT-exon1-43Q and S830 channels on zQ175 images. Recruitment sites indicate the image regions containing recruitment foci that were used for the calculations. The same image regions with pixels in the S830 channel were randomly shuffled, using a custom script, and served as a negative control (shuffled). **b** Pearson correlation coefficient was calculated the same way as in **a** on R6/2 samples. **c** Pearson correlation coefficients were calculated using HTT-exon1-43Q and LAMP1 channels on zQ175 images, with pixels in the LAMP1 channel randomly shuffled to serve as a negative control. Images were taken from cortical, striatal and hippocampal CA1 regions for **a,** hippocampal CA1 region for **b** and **c**. 10-30 images were analysed for each brain region. Statistical analysis was by Welch's two sample *t*-test, ***$p < 0.001$.

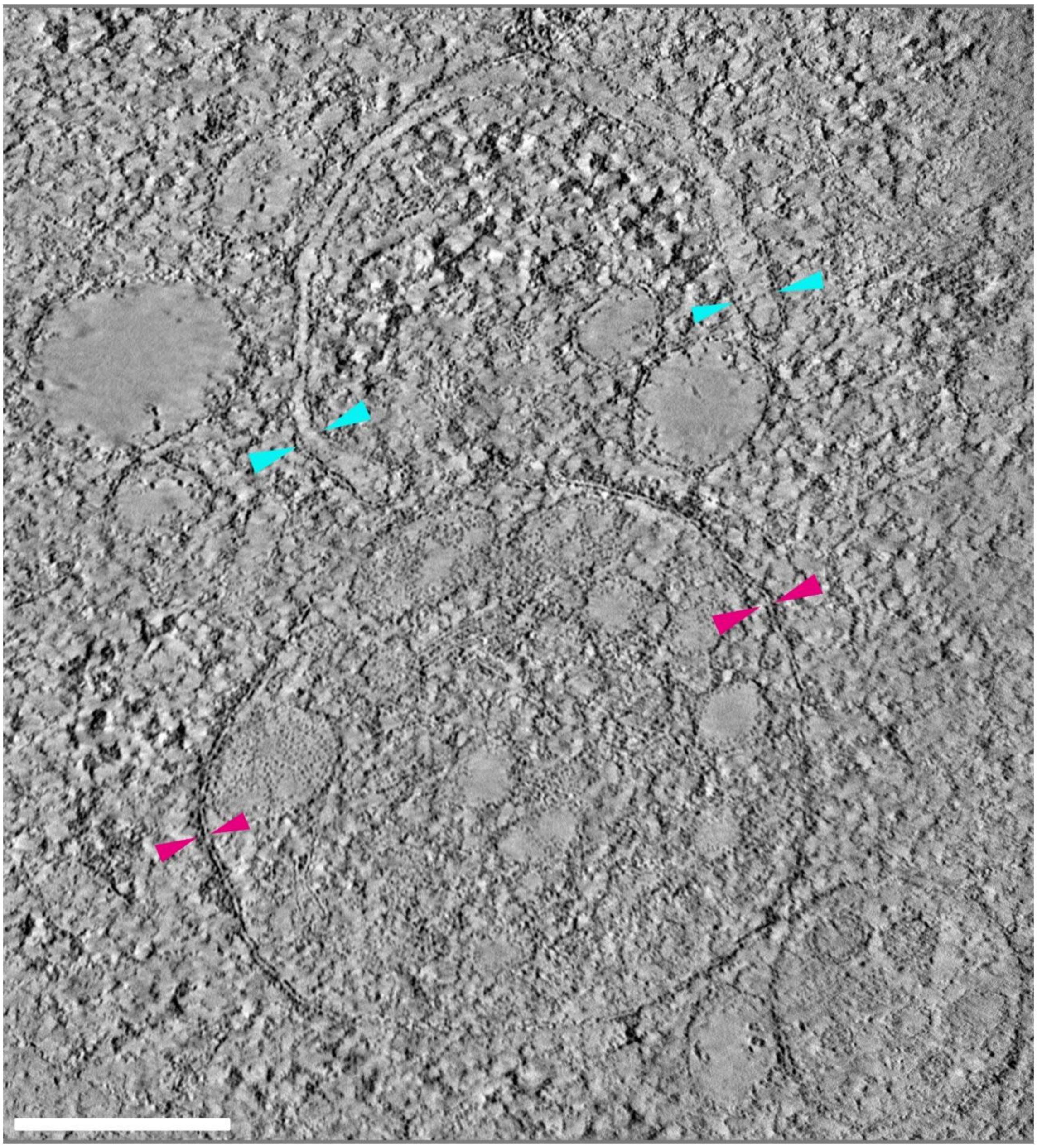

Fig. S3 Full-sized image of the organelle shown in Fig. 4g. Average of 10 central slices from the tomogram acquired for the organelle shown in Fig 4g. The single membrane of the recruitment organelle and double membrane of the nearby phagophore-like structure are indicated by magenta and cyan arrowheads, respectively. The lipid bilayers of the organelle and phagophore-like membranes are clearly visualized. Scale bar: 200 nm.

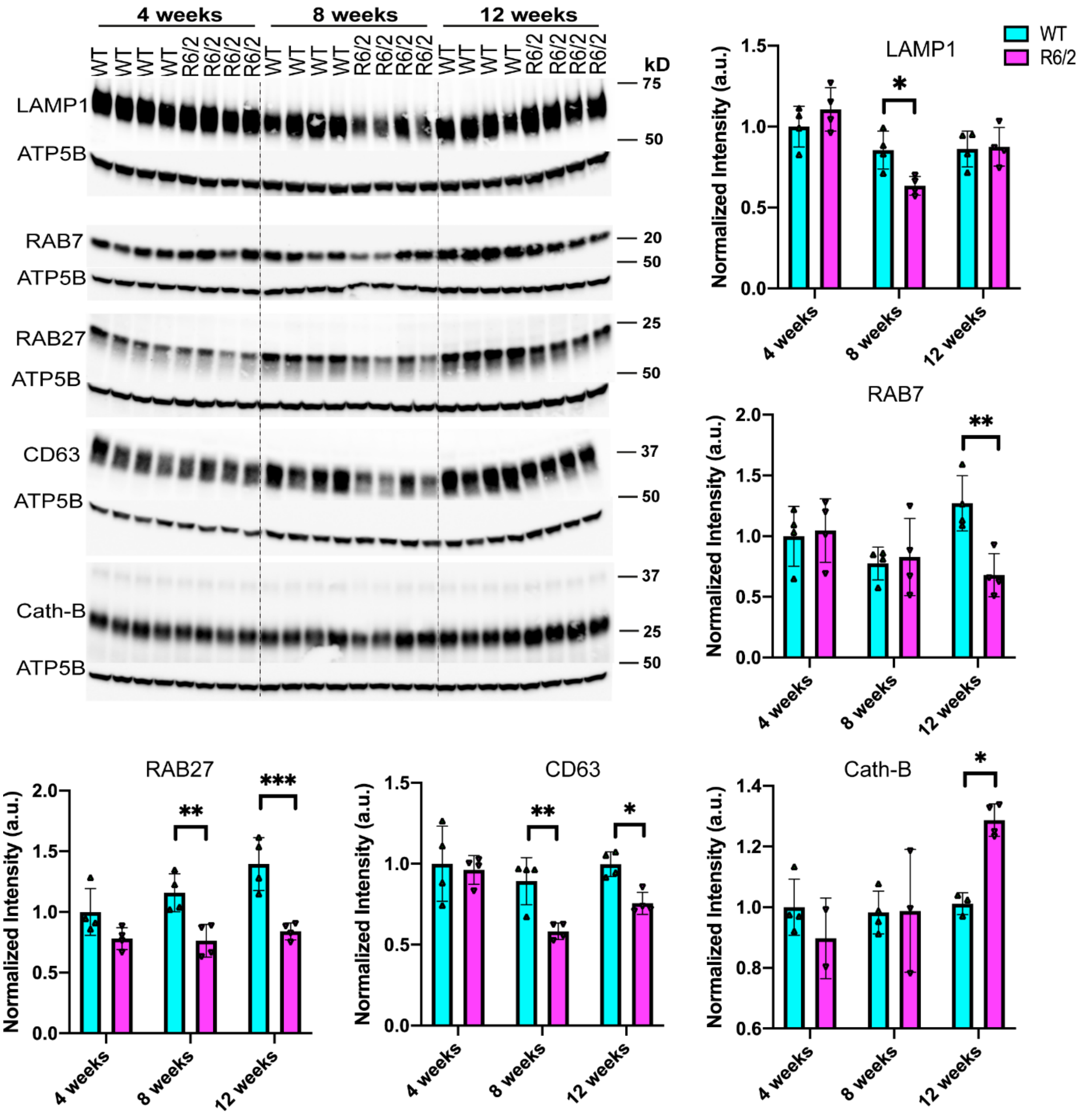

Fig. S4 Western blot of endolysosomal proteins in the R6/2 hippocampus. Western blotting of total protein hippocampal lysates from 4-, 8- and 12-week-old R6/2 mice and their wild-type littermates immunoprobed for either: LAMP1, RAB7, RAB27, CD63 or Cathepsin B (Cath-B) (n = 4 / genotype / age). ATP5B served as loading control. Quantification of the blot is shown on the right and below. The uncropped blots are shown in Supplementary Fig. 4, online resource 1. Statistical analysis was by two-way ANOVA. Data represented by mean ± S.D. *$p<0.05$; **$p < 0.01$; ***$p < 0.001$. Cath-B = Cathepsin B.

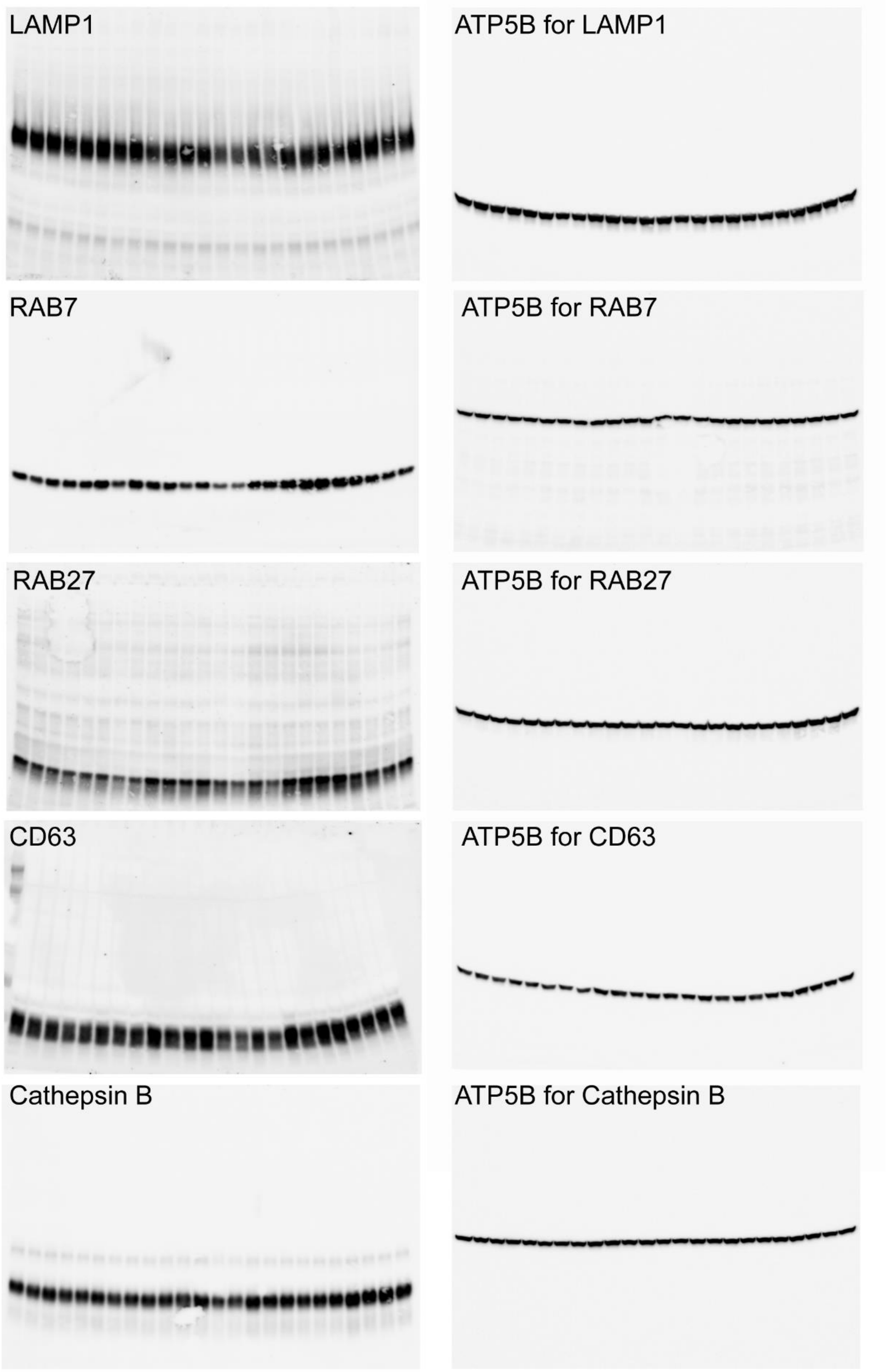

Fig. S5 Full-sized blots for the western blots presented in Supplementary Fig. S4.

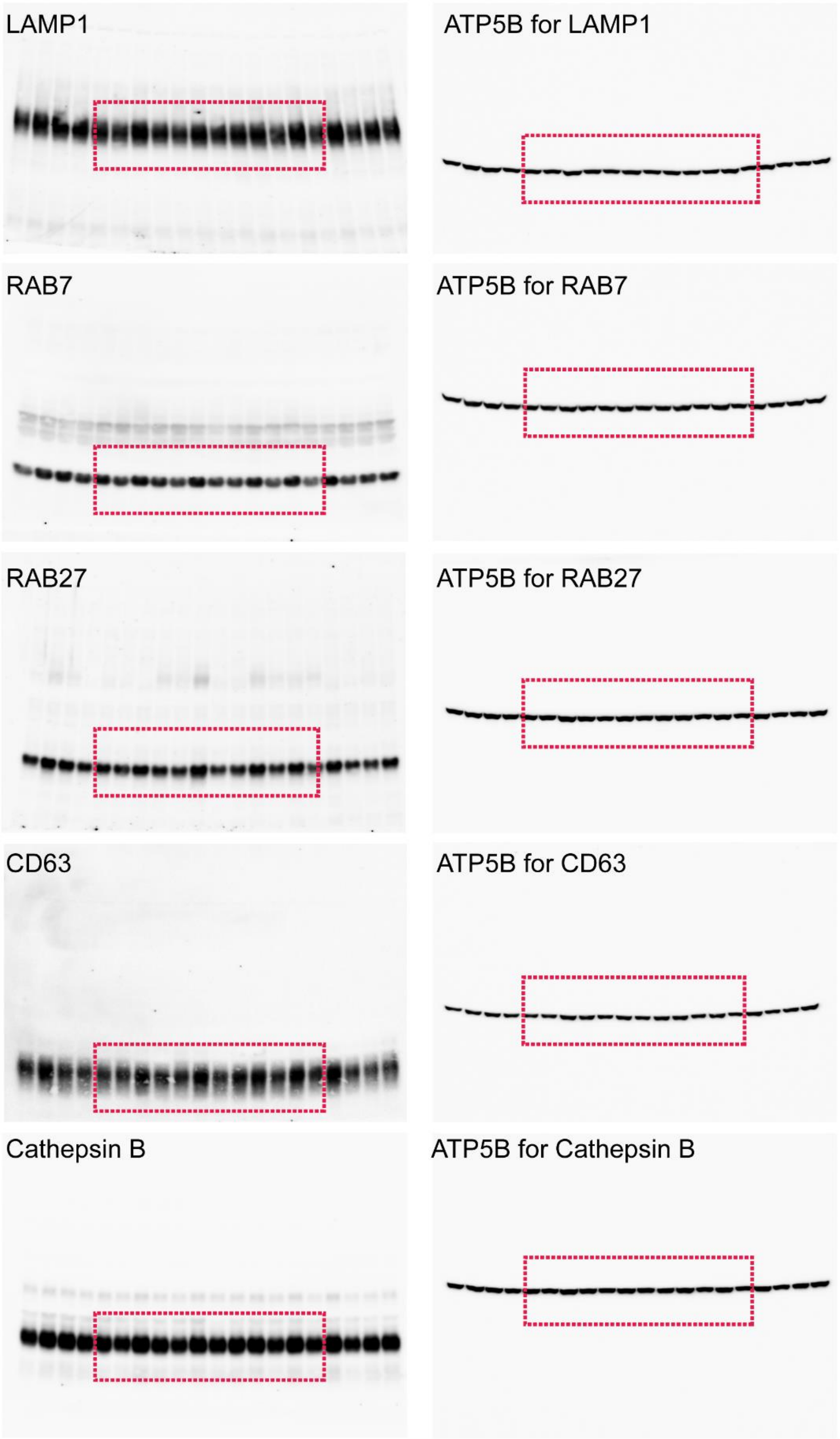

Fig. S6 Full-sized blots for the western blots presented in Fig. 8a. Red rectangular boxes indicate the areas shown in Fig. 8a.

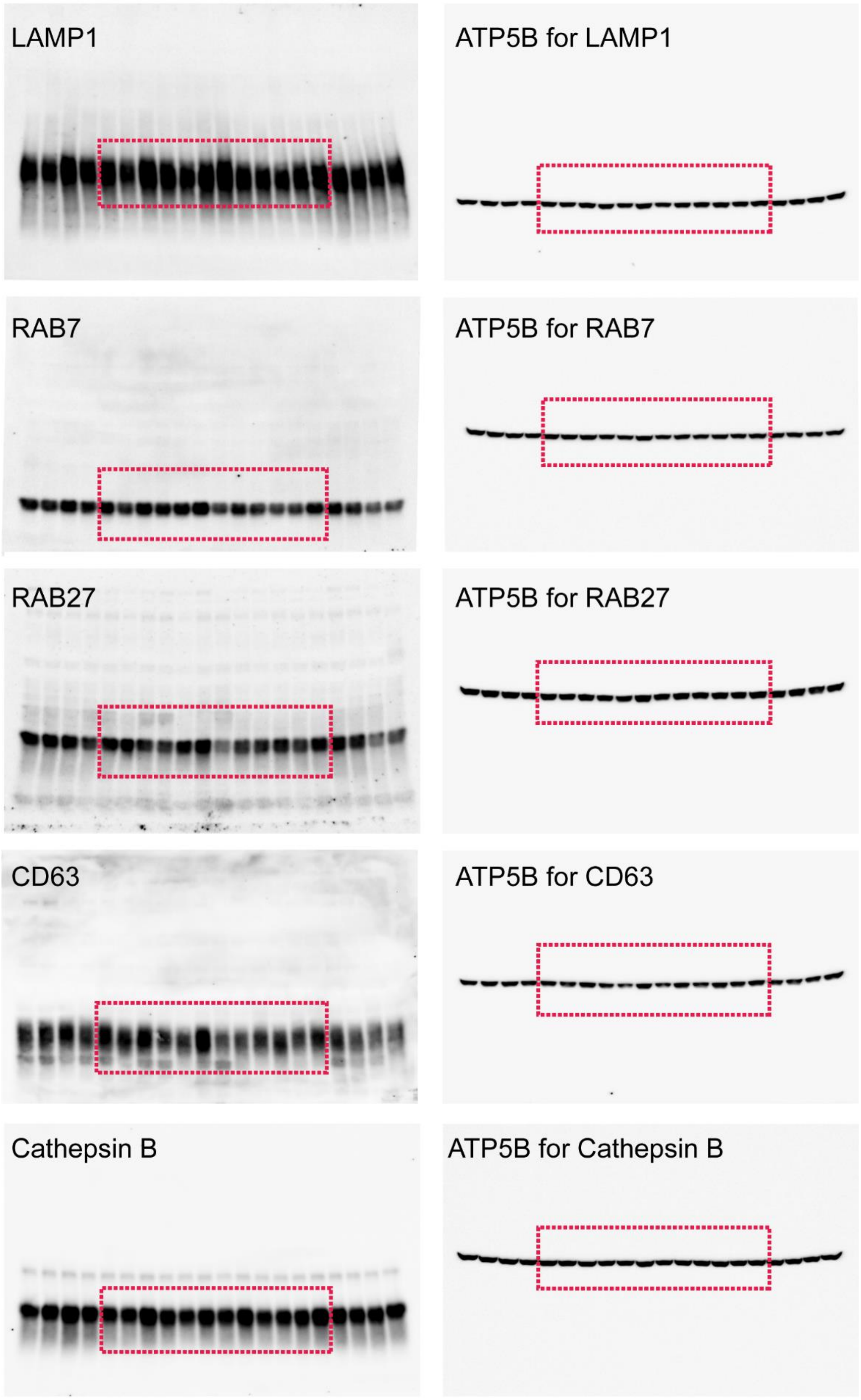

Fig. S7 Full-sized blots for the western blots presented in Fig. 8c. Red rectangular boxes indicate the areas shown in Fig. 8c.

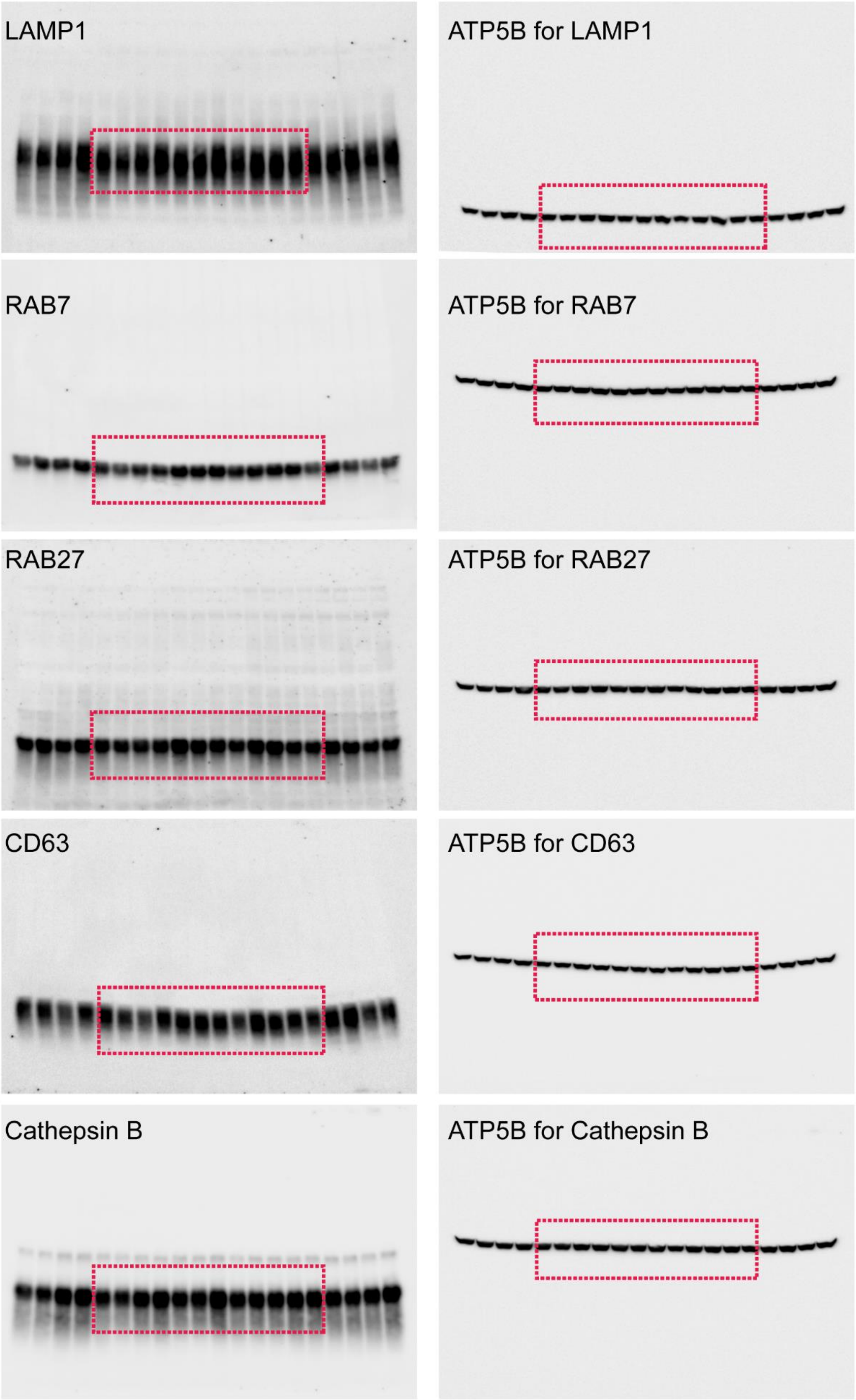

Fig. S8 Full-sized blots for the western blots presented in Fig. 8e. Red rectangular boxes indicate the areas shown in Fig. 8e.

References

1. Sathasivam K, Woodman B, Mahal A, Bertaux F, Wanker EE, Shima DT, Bates GP (2001) Centrosome disorganization in fibroblast cultures derived from R6/2 Huntington's disease (HD) transgenic mice and HD patients. Hum Mol Genet 10:2425-2435. doi:10.1093/hmg/10.21.2425

Supplementary Videos

Additional File 2: Video S1 Tomogram related to Fig. 4g, a recruitment site (segmented in magenta) identified by CLEM in 6-month-old zQ175 mice.

Additional File 3: Video S2 Tomogram related to Fig. 6b, mutant HTT containing organelle resembles MVB/amphisome in 6-month-old zQ175 mice.

Additional File 4: Video S3 Tomogram related to Fig. 6c, mutant HTT containing organelle resembles autolysosome in 6-month-old zQ175 mice. Intraluminal lamellar structures are segmented in cyan.

Additional File 5: Video S4 Tomogram related to Fig. 6d, mutant HTT containing organelle resembles autolysosome/residual body in 6-month-old zQ175 mice.
